# The extracellular matrix of ovarian cortical inclusion cysts modulates invasion of fallopian tube epithelial cells

**DOI:** 10.1063/1.5022595

**Published:** 2018-04-11

**Authors:** Andrew J. Fleszar, Alyssa Walker, Veronica Porubsky, Will Flanigan, Darian James, Paul J. Campagnola, Paul S. Weisman, Pamela K. Kreeger

**Affiliations:** 1 Department of Biomedical Engineering, University of Wisconsin-Madison, Madison, Wisconsin 53705, USA; 2 University of Wisconsin Carbone Cancer Center, University of Wisconsin School of Medicine and Public Health, Madison, Wisconsin 53705, USA; 3 Department of Pathology, University of Wisconsin School of Medicine and Public Health, Madison, Wisconsin 53705, USA; 4 Department of Obstetrics and Gynecology, University of Wisconsin School of Medicine and Public Health, Madison, Wisconsin 53705, USA; 5 Department of Cell and Regenerative Biology, University of Wisconsin School of Medicine and Public Health, Madison, Wisconsin 53705, USA

## Abstract

A growing body of research supports the idea that the fallopian tube epithelium (FTE) is the precursor for most high-grade serous ovarian cancers (HGSOCs) but that the ovary plays a critical role in tumor metastasis. Cortical inclusion cysts (CICs) in the ovarian cortex have been hypothesized to create a niche environment that plays a role in HGSOC progression. Through histological analysis of pathology samples from human ovaries, we determined that collagen I and III were elevated near CICs and that the collagen fibers in this dense region were oriented parallel to the cyst boundary. Using this information from human samples as design parameters, we engineered an *in vitro* model that recreates the size, shape, and extracellular matrix properties of CICs. We found that FTE cells within our model underwent robust invasion that was responsive to stimulation with follicular fluid, while ovarian surface epithelial cells, the native cells of the ovary, were not invasive. We provide experimental evidence to support a role of the extracellular matrix in modulating FTE cell invasion, as a decrease in collagen I concentration or the addition of collagen III to the matrix surrounding FTE cells increased FTE cell invasion. Taken together, we show that an *in vitro* model of CICs obtained from the analysis of human tissue can act as an important tool for understanding FTE cell interactions with their environment.

## INTRODUCTION

**FIG. 1.**
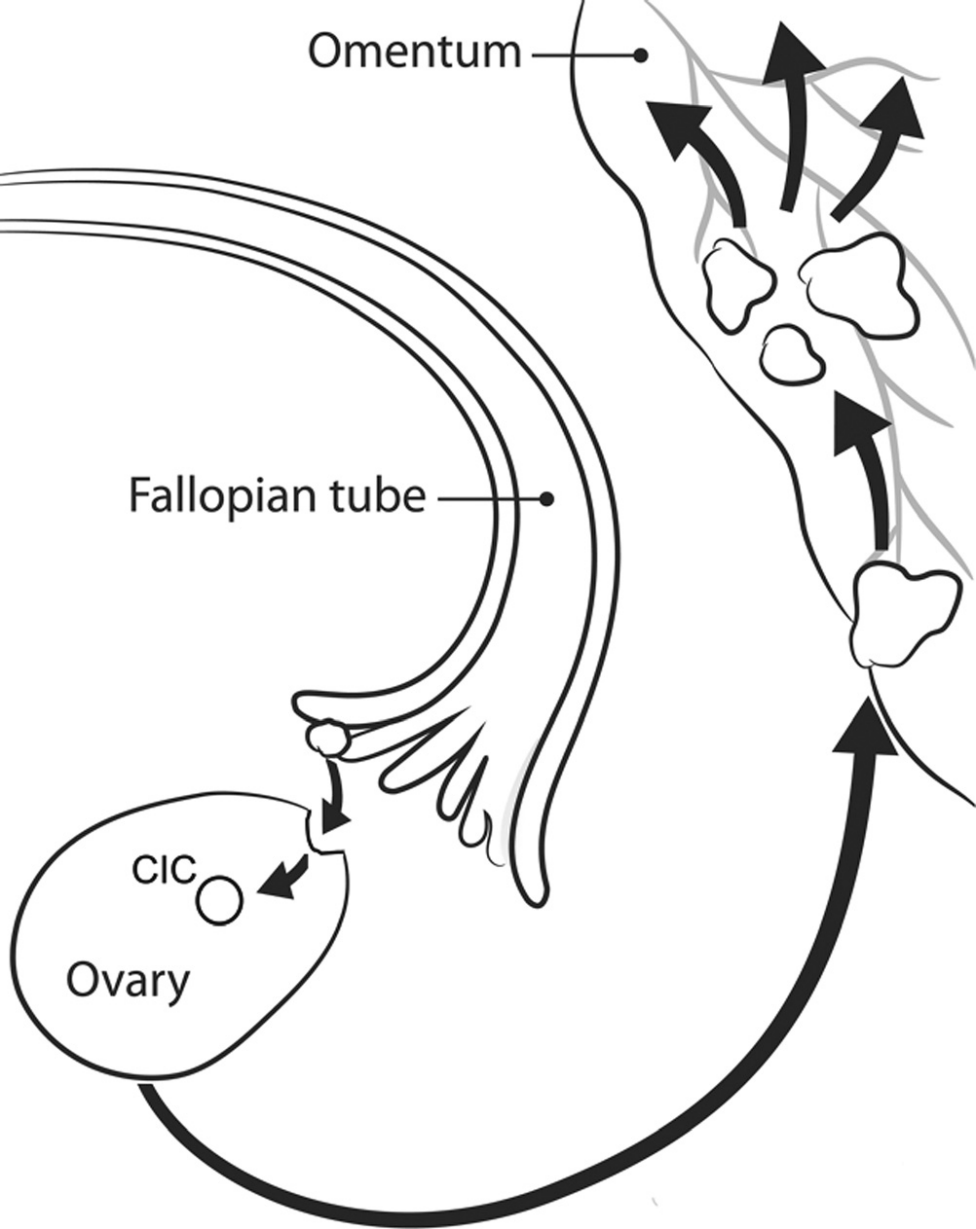
The hypothesized progression of HGSOC. FTE cells on the fallopian tube fimbria are exfoliated and embed in CICs within the ovary. The niche environment of these cysts causes the cancerous progression of FTE cells to HGSOC tumors that spread throughout the peritoneal cavity. Modified from the original artwork by Jennifer Zernick.

High-grade serous ovarian cancer (HGSOC) has a 10-year survival rate of less than 30%, a prognosis that has not significantly improved over the past 30 years.^1^ As a result of the field's limited understanding of the early stages of disease progression, there are currently no reliable screening methods for HGSOC and over 70% of patients are diagnosed with stage III or IV tumors that have metastasized beyond the pelvis.^2^ For many years, it was thought that HGSOC originated from ovarian surface epithelial (OSE) cells; however, recent evidence suggests that HGSOC begins on the distal end of the fallopian tube^3–5^ (Fig. 1). In this new paradigm, the fallopian tube epithelium (FTE) acquires *TP53* mutations and undergoes malignant transformation to form precursor lesions known as serous tubal intraepithelial carcinomas (STICs).^6^

Although HGSOC likely originates on the fallopian tube, evidence suggests that the ovary still plays an important role. Epidemiologically, a woman's number of lifetime ovulations is correlated with increased risk of ovarian cancer, and birth control and multiple pregnancies are potential preventative measures.^7^ A mouse model of HGSOC that originated from the PAX8-positive cells of the fallopian tube showed that, while lesions on the fallopian tube developed following oophorectomy, there was a significant reduction in peritoneal metastasis without the ovary.^8^ Furthermore, a mouse model of HGSOC has shown that the ovulatory wound promotes the migration and adhesion of malignant cells to the ovary, potentially initiating the metastatic cascade.^3^

One distinct feature of the ovary associated with ovulation, which has long been thought to play a role in the progression of ovarian cancers, is the cortical inclusion cyst (CIC).^9^ CICs are spherical, epithelial-lined cysts (<1 cm) found within the cortex of the ovary (Fig. 1). These cysts are thought to form as a result of ovulation wounds to the ovarian surface as well as age-related surface invaginations.^10^ OSE cells migrate into these cysts as they form, and it was previously hypothesized that the unique microenvironment of these cysts induced the transformation of OSE cells to an invasive carcinoma.^11,12^ With recent evidence suggesting that the FTE cells, rather than OSE cells, are the precursors for HGSOC, researchers have begun to revisit the role that CICs play in HGSOC development. Analysis of pathological sections has revealed that both FTE cells and epithelial tumor precursors can be present in ovarian CICs, indicating that there is still a potential link between these cysts and HGSOC development.^13^

Therefore, we sought to investigate the physical and chemical elements of the CIC in order to address the hypothesis that the CIC microenvironment enhances metastasis of FTE cells. The tumor microenvironment is known to influence metastasis in breast cancer, as differences in the composition and physical organization of extracellular matrix (ECM) proteins near tumors can initiate and direct cell invasion.^14–16^ In the ovary, collagen I and collagen III are the main structural elements of the ECM, but these proteins are in flux due to the extensive ECM remodeling associated with follicle maturation and ovulation.^17,18^ This turnover can shift the balance of ECM production and degradation and may give CICs a unique ECM composition and structure compared to other parts of the ovary. However, the levels of collagen I and collagen III in the ovarian cortex and CICs have not been reported. Therefore, we first characterized the levels of these proteins and then utilized this information to design a novel *in vitro* model of the CIC where perturbations to the CIC microenvironment could be examined.

## RESULTS

### CIC boundaries contain a dense band of collagen

**FIG. 2.**
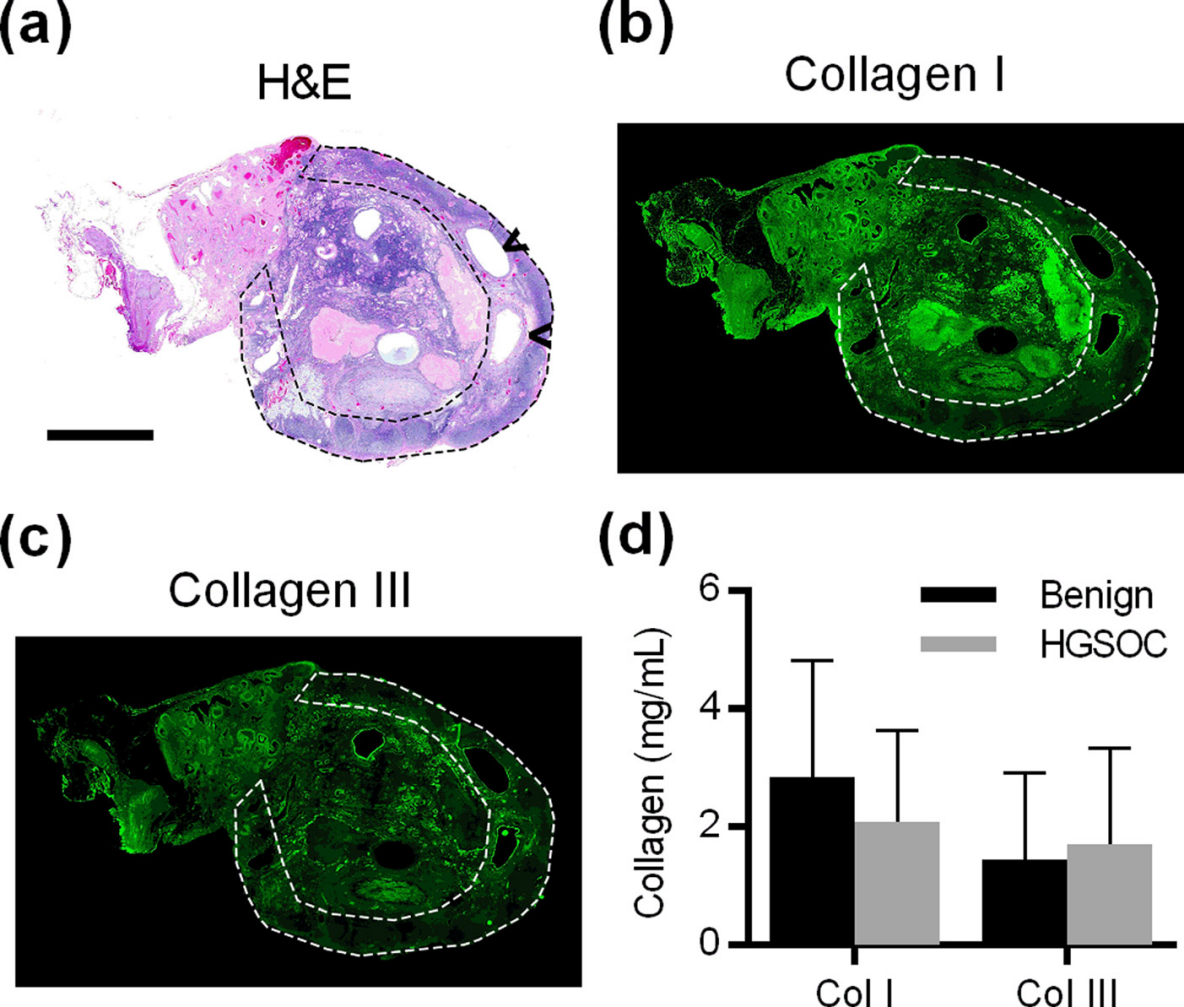
(a) H&E stain of human ovary; arrowheads indicate the CICs, and dotted line highlights the cortex (Scale bar* *= 5 mm). [(b), (c)] IMF showing the distribution of collagen I and III in the ovary and cortex for the same ovary as in (a). (d) Quantification of collagen I and III in the ovarian cortex, n* *= 10 per condition, not significantly different by the two-sided t-test.

Paraffin embedded ovary sections from 10 patients with HGSOC and 10 patients that had their ovaries removed for benign conditions (supplementary material, Table 1) were stained with hematoxylin and eosin (H&E) to identify the cortex, where follicles and CICs reside [Fig. 2(a)]. Collagen I and collagen III are the main structural components of the ovary^17^ and have been known to influence metastasis in other tumor types. The distribution of these ECM proteins in the ovary was visualized by immunofluorescence (IMF) staining [Figs. 2(b) and 2(c)]. From these images, the average fluorescence signal in the cortex was compared with the signal from the whole ovary to obtain the relative level of collagen in the cortex. These relative levels were combined with the absolute level of collagen I and collagen III measured via dot blot (supplementary material, Fig. S1) to obtain the average collagen I and collagen III concentrations in the ovarian cortex [Fig. 2(d)]. The concentration of collagen I in the cortex was determined to be approximately 2.9 mg/ml for benign patients and 2.1 mg/ml for HGSOC patients. The concentration of collagen III in the cortex was found to be approximately 1.5 mg/ml for benign patients and 1.7 mg/ml for HGSOC patients. These concentrations are comparable to the levels seen in other non-structural tissues.^19^ Neither collagen I nor collagen III concentrations were significantly different between patients with HGSOC and patients that had their ovaries removed for benign conditions.

**FIG. 3.**
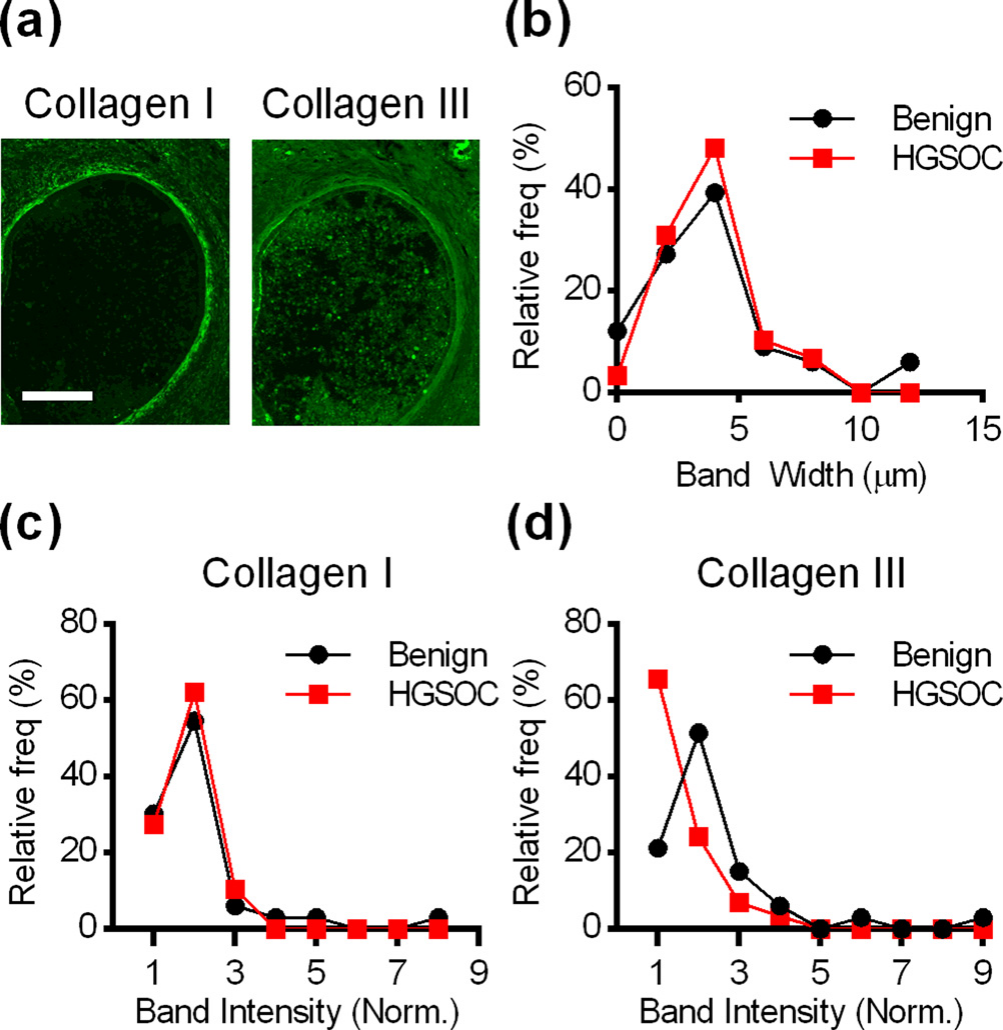
(a) IMF for collagen I and III near CICs revealed a dense band of collagen surrounding CICs (Scale bar* *= 50 *μ*m). (b) Histogram of the width of the collagen I band for benign and HGSOC patients. [(c), (d)] Histograms showing the relative fluorescence intensity of the collagen I and III bands compared to stroma near the cyst. For [(b)–(d)], data are presented as frequency distributions from 33 CICs from 10 patients with benign conditions and 29 CICs from 10 patients with HGSOC.

While there were no patient-population based differences in ovarian collagen I or collagen III concentrations, IMF demonstrated that there was an uneven distribution of collagens throughout the ovary. In particular, some parts of the ovary, including blood vessels (supplementary material, Fig. S2) and CICs [Fig. 3(a)], had dense patterns of collagen adjacent to their lumens. To examine this quantitatively, 62 small cysts lined with epithelial cells and contained within the ovary cortex were identified in ovarian sections (1–9 CICs/ovary). Image analysis showed that CICs were surrounded by a dense collagen band with an average thickness of approximately 4 *μ*m as measured from collagen I images [Fig. 3(b)] and a fluorescence intensity approximately twice that of the cortex stroma [Figs. 3(c) and 3(d)]. Student's t-tests showed that the average bandwidth and fluorescence intensity were not significantly different in patients with benign conditions and patients with HGSOC.

### The collagen band in CICs is aligned parallel to the CIC boundary

**FIG. 4.**
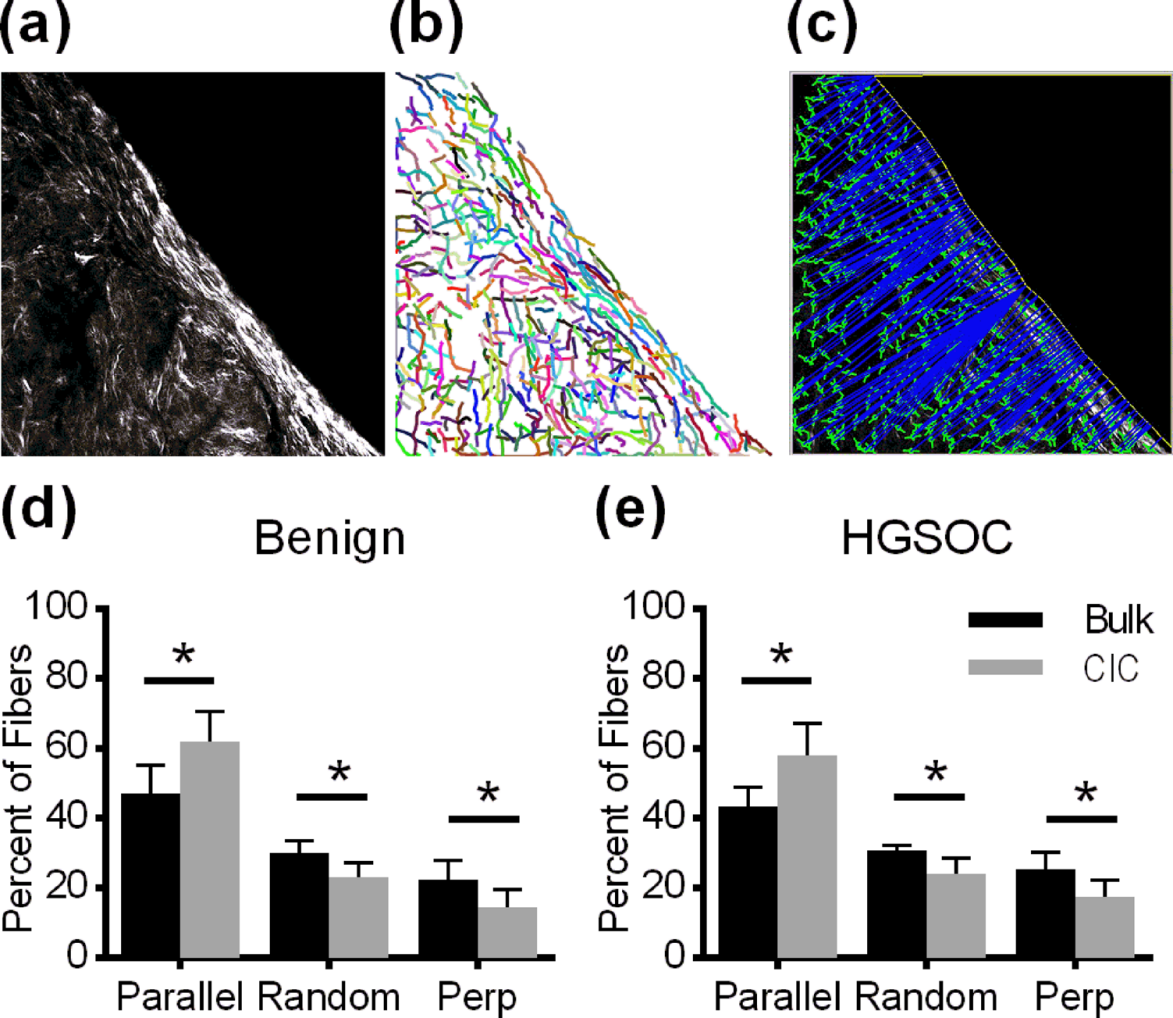
(a) Collagen Specific Second Harmonic Generation image of collagen at the CIC boundary (Scale bar* *= 20 *μ*m). (b) CT-FIRE identification of collagen fibers. (c) CurveAlign image identifying the angle and distance from the boundary for each fiber. [(d)–(e)] Collagen fibers at the CIC boundary were significantly more aligned to the boundary compared to stromal fibers in both benign (n = 10) and HGSOC (n = 13) patients. Data are an average of the alignment percentages found in each CIC, n* *= 904–6544 fibers per CIC, and *p *< 0.001 between the CIC boundary and bulk by a two-sided t-test.

In contrast to the ECM composition and concentration, which could potentially inhibit or promote cell invasion, collagen alignment has been shown to increase the persistence of cell invasion in the direction of alignment.^16^ To determine if the collagen band surrounding CICs has a unique alignment signature, Collagen Specific Second Harmonic Generation (SHG) Imaging was used to visualize the collagen fiber structure at the CIC boundary. This imaging technique takes advantage of the nonlinearity of collagen fibers to visualize their orientation within a matrix.^20^ Z-stacks of the collagen band surrounding each CIC were taken [Fig. 4(a)], and the images from these z-stacks were run through an algorithm designed to extract individual collagen fibers from images [Fig. 4(b)].^21^ Using this algorithm, we quantified the fiber location and orientation and then determined the distance and orientation of each fiber relative to a manually drawn line reflecting the CIC boundary [Fig. 4(c)]. Collagen fibers within 10 *μ*m of the CIC boundary were considered part of the collagen band surrounding CICs since this distance captured the entirety of the collagen band in over 90% of CICs [Fig. 3(b)]. Fibers further than 10 *μ*m from the CIC boundary were considered part of the bulk stroma. Fibers in the boundary region contained a significantly higher percentage of fibers parallel to the boundary than that was found in the bulk stroma [Figs. 4(d) and 4(e)]. While there are differences in collagen fiber alignment between the boundary region and the bulk stroma, student's t-tests revealed that there are no significant differences between patients with benign conditions and patients with HGSOC.

### *In vitro* model of CICs

**FIG. 5.**
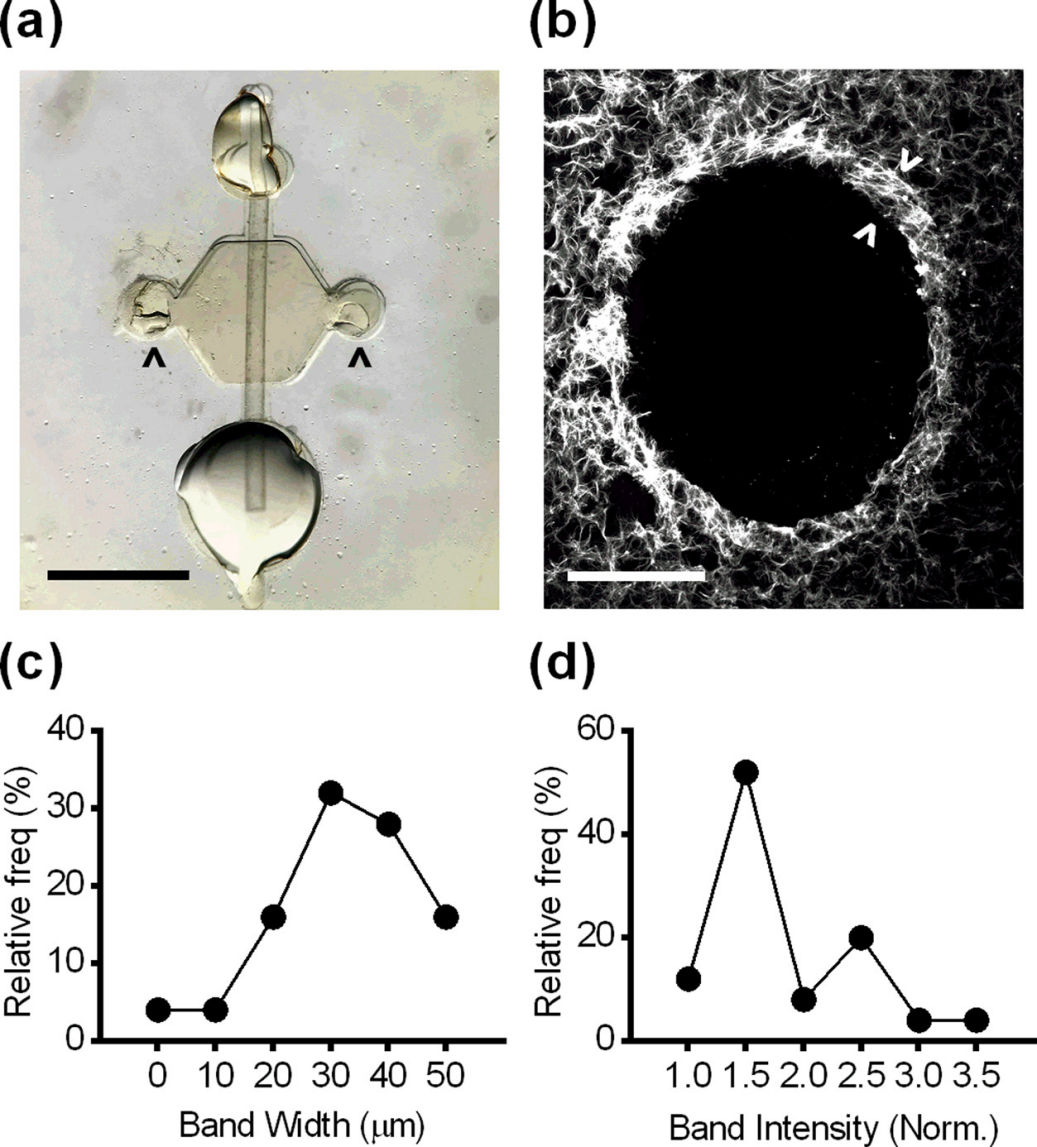
(a) Microfluidic lumen with a small port (top), a large port (bottom), side ports (arrowheads), and a lumen rod (Scale bar* *= 2 mm). (b) Collagen formed a dense band (arrowheads) surrounding lumens in the *in vitro* model (Scale bar* *= 200 *μ*m). (c) Histogram of the width of the collagen band. (d) Histogram of the normalized fluorescence intensity of the collagen band. For [(c) and (d)], data are presented as frequency distributions, n* *= 25 lumens.

Due to the buried location of CICs in the ovary and randomness of their formation, it is not possible to follow the progression of FTE cells in CICs in *in vivo* models. Additionally, *in vitro* models provide flexibility to examine the impact of many physical and chemical characteristics on cellular behaviors. Therefore, we modified a microfluidic platform^22^ to recapitulate the CIC microenvironment. The model consists of a cylindrical lumen embedded within an ECM hydrogel [Fig. 5(a)], where the diameter of the lumen and the composition of the ECM hydrogel could be altered to reflect CIC characteristics. To replicate the *in vivo* CIC environment as closely as possible, the lumen diameter was set near the average diameter measured from the 62 CICs found in human ovaries (430 *μ*m, supplementary material, Fig. S3). Although CICs are generally spherical, this cylindrical model provided a close approximation of local curvature and surface area-to-volume ratio, while enabling cells and different soluble stimuli to be added to the model. For our initial optimization, we utilized collagen I alone as it is the most prevalent ECM protein based on our measurements from human ovary sections (Figs. 2 and 3). To examine the ECM in the model, lumens were stained with a fluorescent collagen-binding protein, cryosectioned, and visualized. From this analysis, we determined that the lumens retained their size and shape and were surrounded by a dense band of collagen similar to the collagen I signature of human CICs [Fig. 5(b)]. This dense band was observed in lumens made from a broad range of collagen concentrations. Upon quantification, the width of the collagen band [Fig. 5(c)] was found to be significantly higher, but within an order of magnitude of the band measured in human CICs. The normalized fluorescence intensity [Fig. 5(d)] was not statistically different from the pattern measured in human CICs (supplementary material, Fig. S4).

### FTE cells undergo robust invasion in an *in vitro* CIC model and respond to follicular fluid stimulation

**FIG. 6.**
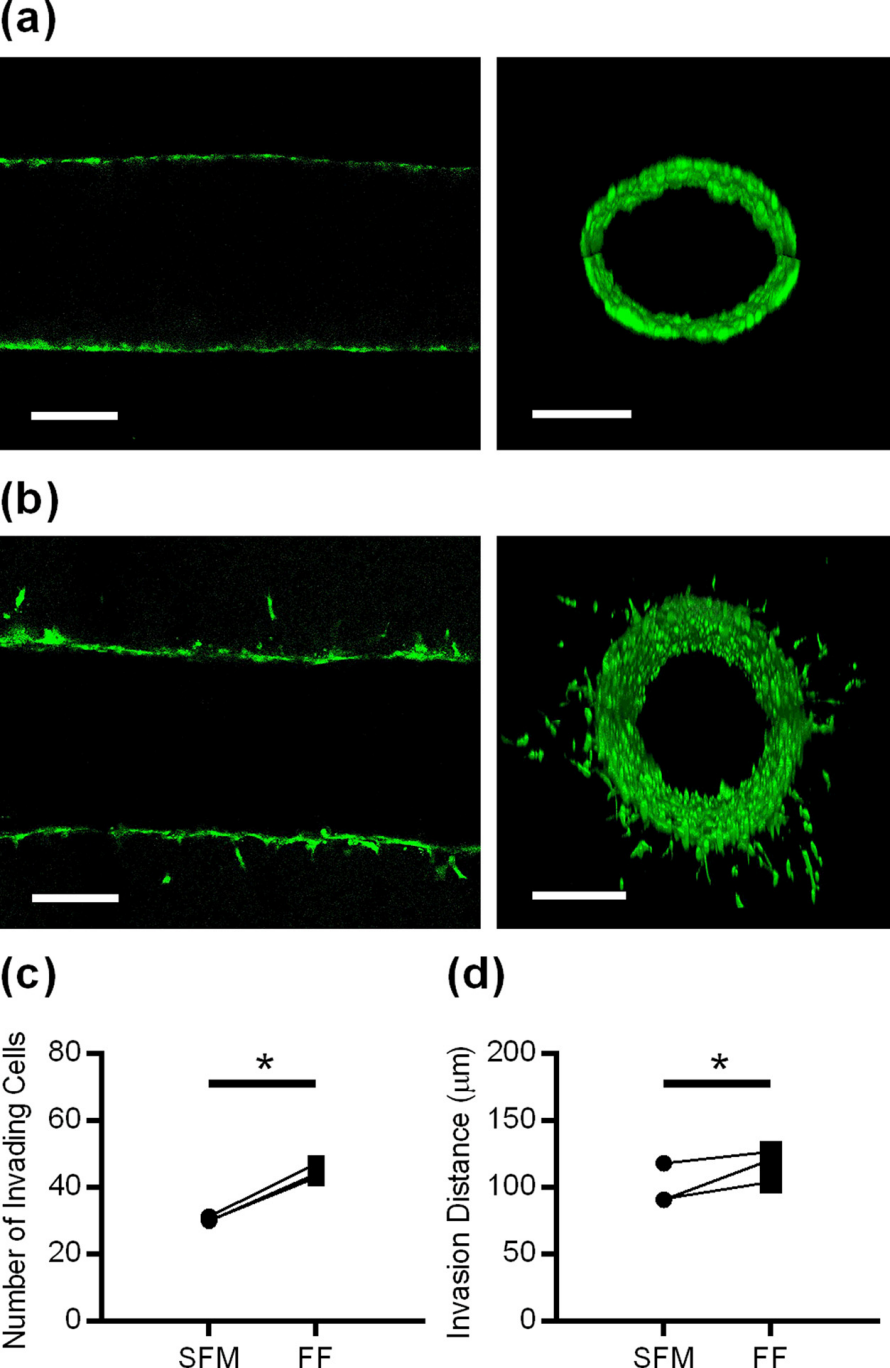
(a) Confocal slice and Z-stack cross-section reconstruction of CellTracker™ Green-labeled FTE cells in the *in vitro* CIC model two hours after seeding. (b) Confocal slice and Z-stack cross-section reconstruction of FTE cells after 48 h in lumen, demonstrating that individual cells migrated into the ECM from the confluent layer (scale bar* *= 200 *μ*m). [(c) and (d)] Stimulating FTE cells with follicular fluid increased the number of cells that invade and invasion distance. Data in [(c) and (d)] are shown as the average per patient sample, n* *= 3 individual patients with 10–13 lumens/patient. * indicates *p *< 0.001 for (c) and *p *< 0.01 for (d) using the paired t-test.

The entrapment of FTE cells within CICs^13^ was mimicked by seeding mouse FTE cells with a *Tp53* mutation into our *in vitro* CIC model. Confocal microscopy showed that FTE cells formed a confluent monolayer around the entire lumen after the initial attachment period [Fig. 6(a)]. Over the next 48 h, FTE cells underwent robust invasion radially out of the lumen into the surrounding ECM [Fig. 6(b)]. The pattern of invasion resembled either mesenchymal or amoeboid migration,^23^ as single cells broke away from the collective as they invaded out of the lumen. It is possible that secondary cells followed leader cells, as we often observed multiple cells in a line coming from the center lumen. In stark contrast to FTE cells, primary mouse OSE cells seeded in the CIC model did not undergo any invasion over 48 h (supplementary material, Fig. S4). To validate the efficacy of our model, we wanted to ensure that FTE cells seeded in microfluidic lumens would respond to chemical stimuli. We chose to stimulate cells with follicular fluid since this fluid contains a diverse and highly concentrated collection of pro-inflammatory cytokines and has been hypothesized to play an important role in HGSOC initiation.^24^ When FTE cells in the *in vitro* CIC model were stimulated with follicular fluid, there was a significant increase in the number of FTE cells that invaded [Fig. 6(c)] and the distance of invasion for all donor samples [Fig. 6(d)]. While follicular fluid stimulation from different donors consistently increased invasion, both the number of invading cells (1.40-fold to 1.51-fold) and the average invasion distance (1.07-fold to 1.33-fold) varied slightly from patient-to-patient.

### The physical and chemical microenvironments of CICs affect FTE cell invasion

**FIG. 7.**
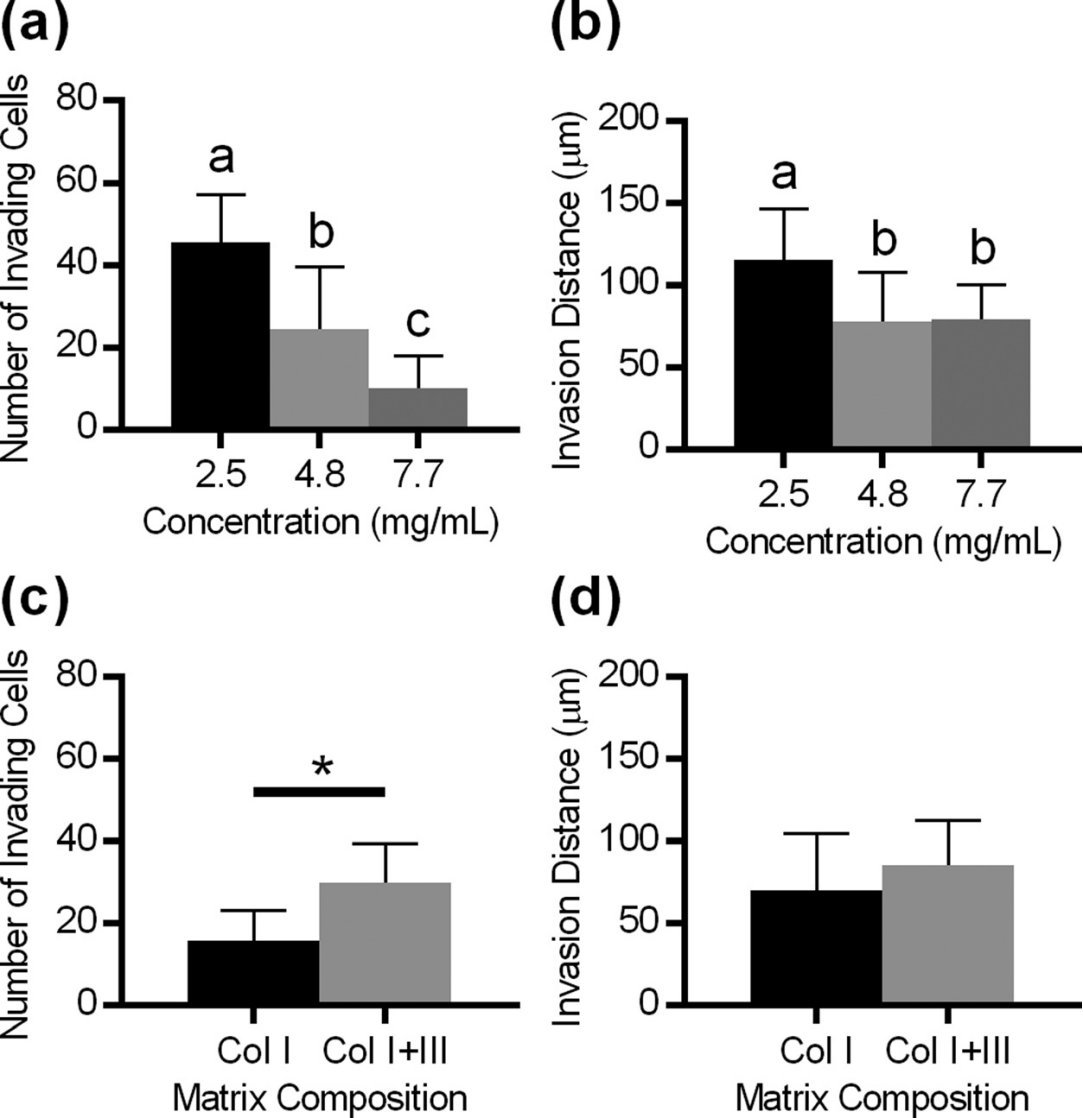
[(a) and (b)] Increasing collagen density decreased the number of cells that invaded and the distance of invasion. n* *= 12–43 lumens per condition, different letters indicate significantly different groups by Tukey-HSD, and *p *< 0.01. [(c), (d)] Incorporating collagen III increased the number of cells that invaded but did not impact the invasion distance. n* *= 17–19 lumens per condition; * indicates *p *< 0.001 by the two-sided t-test.

A key advantage of *in vitro* biomimetic models is that they allow the impact of microenvironmental parameters (e.g., ECM concentration and composition) on cell behavior to be easily examined and quantified. Based on our observation of relatively high variability of the collagen I concentration in the ovarian cortex (CV = 71%), the concentration of collagen I in the hydrogel was adjusted between 2.5 mg/ml and 7.7 mg/ml, which is within the physiological range obtained from ovarian samples [Fig. 2(d)]. The increase in the collagen I concentration significantly reduced both the number of FTE cells that invaded [Fig. 7(a)] and the distance of invasion [Fig. 7(b)] in the CIC model. Since we had observed the presence of collagen III in both the ovarian cortex and the band surrounding the CIC, we next examined FTE cell invasion into a matrix containing both collagen I and collagen III, where the total protein content remained constant. Collagen III in the matrix significantly increased the number of cells that invaded [Fig. 7(c)] but did not impact the average distance of invasion [Fig. 7(d)]. Taken together, these data support that the ECM properties surrounding CICs can alter the invasive characteristics of FTE cells.

## DISCUSSION

Recent development of the “tubal hypothesis” for the origin of HGSOC^5,6^ has called into question the role of the ovary in this subtype of ovarian cancer. Evidence from animal models continues to support a role for the ovary in progression to advanced HGSOC,^3,8^ and pathological analysis has demonstrated the presence of cells resembling the putative precursor cell, the FTE, in CICs.^13^ However, analyzing progression in the CIC is complicated by the inability to visualize and monitor CICs *in vivo.* The *in vitro* CIC model that we developed offers two significant advantages over animal models: (1) FTE cell behavior within this model can be analyzed in real time and (2) the components that make up this model can be modulated individually or in combination to determine how these variables impact FTE cell behavior.

To ensure that our model was based on physiologically relevant conditions, we first undertook a detailed characterization of the physical and chemical composition of the ECM surrounding CICs in human ovaries. Using our characterization as design parameters, we developed a CIC model that reflected the average CIC size and collagen I concentrations and examined the behavior of the two cell types found within CICs—the OSE and the FTE.^13^ While both cell types were able to form a confluent layer covering the lumen wall, drastically different behaviors were observed over the time period examined. FTE cells with a *Tp53* mutation (the hypothesized precursors to HGSOC) underwent spontaneous and robust invasion into the surrounding ECM, while OSE cells (the native cells from the ovary) did not invade from the lumen. To our knowledge, this is the first time a phenotypical difference between FTE and OSE cells has been demonstrated *in vitro*. While this result may relate to the presence of the *Tp53* mutation, this is physiologically relevant for the initiation of ovarian cancer. There are numerous studies showing that *Tp53* mutations occur on the fallopian tube as one of the first steps in HGSOC progression;^6,25^ however, there is no evidence showing that *Tp53* mutations occur on the ovarian surface.^12^ Over 90% of HGSOC tumors have an alteration in *TP53.*^26^ Based on these observations, we infer that FTE cells that land in CICs and progress to tumors are likely to have a *Tp53* mutation, while any OSE cells that are incorporated into CICs will not have this mutation.

This contrasting behavior supports the hypothesis that the implantation of FTE in the ovary is an important step to develop advanced HGSOC tumors.^27^ The wound remaining after ovulation is a likely site of FTE cell implantation, and studies have demonstrated that FTE cells preferentially adhere to the wounded ovaries compared to intact ovaries.^28^ The pro-inflammatory follicular fluid milieu that is released during ovulation has been shown to promote enhanced migration and preferential adhesion of FTE cells to the ovarian stroma.^3^ To further investigate the behavior of FTE in our *in vitro* CIC environment, we examined the cells' response to the soluble stimuli found in follicular fluid and confirmed that FTE cells seeded within our *in vitro* model were responsive to chemical stimuli that would be present in the CIC *in vivo*.

The ECM plays a prominent role in ovarian structure and function, with collagen I and collagen III comprising the main structural components of the ovary.^17^ Previous studies investigating these fibrillar collagens in the developing ovarian follicle found that collagen I and collagen III are organized into concentric layers surrounding follicles and also throughout the ovarian stroma.^17,29^ Furthermore, extreme changes in the ECM surrounding follicles have been observed throughout follicle development and extensive ECM degradation and synthesis are necessary for follicle maturation and ovulation.^17,30^ We examined the local microenvironment of the cortex to determine CIC collagen levels, information that has not been previously reported. The elevated levels of collagen I and collagen III near CICs are perhaps not surprising since these cysts form in conjunction with the enhanced ECM remodeling that takes place during and after ovulation.^31^ The levels of collagen I and III surrounding CICs were not different between normal ovaries and those with tumors, suggesting that ECM changes associated with HGSOC occur following metastasis, rather than predisposing the ovary to colonization.

Our IMF staining and quantitative results were focused on fibrous collagens that support cells and structures such as CICs in the ovary. To further examine the fibrillar structure of CICs, we used Collagen Specific SHG to visualize individual collagen fibers. The pattern of collagen fiber alignment in the stroma changes drastically with the onset of HGSOC, and SHG imaging has been identified as a useful tool for differentiating normal ovarian tissue from HGSOC tumors.^32^ Our analysis demonstrated that in addition to having an increased density, the fibers in the band of collagen surrounding CICs were aligned parallel to the CIC boundary at significantly higher levels than fibers in the stroma bulk. The presence of fibrous collagen has been found to be necessary for the invasion of breast cancer cells, and fibers regulated the extent of the MDA-MB-231 breast cancer cell invasion independent of total ECM concentration.^33^ Importantly, fiber alignment has been shown to provide cues that cells respond to. *In vitro* models of aligned fibers have revealed that while fiber alignment does not affect the overall motility of cells, alignment restricts cell motility largely to one dimension.^34^ Aligned fibers result in increased directional persistence of cells along the fiber direction and limit cell protrusions perpendicular to this alignment, leading to increased migrational efficiency.^16^ Notably, the presence of collagen bundles aligned perpendicular to tumors has been shown to be correlated with poor patient prognosis in breast cancer.^35^ Based on these observations, we would expect that collagen fibers aligned parallel to the CIC boundary likely slow invasion into the stroma.

Since FTE cells undergo extensive invasion in our *in vitro* CIC model at collagen concentrations reflective of average levels of collagen I *in vivo*, we sought to examine the impact of changes in the ECM concentration. The collagen concentration has been shown to be a key mediator of tumor progression in other tumors. For example, a mouse model with increased stromal collagen in mammary tissue showed that enhanced re-organization in collagen-dense tissues facilitated significantly more invasive breast carcinomas.^14^ Importantly, as collagen density increases, the bioavailability of collagen ligands increases, promoting the formation of focal adhesions,^36^ which allows cells to reorganize the ECM and activate downstream signaling pathways modulating cell behaviors such as invasion.^37^ However, the impact of collagen density is not straightforward as increased collagen density has also been shown to act as a passive barrier to cell invasion unless degraded by matrix metalloproteinases.^38^ In our *in vitro* CIC model, we observed an inverse relationship, with FTE cell invasion increasing in lower collagen I concentrations. This behavior may help to explain why HGSOC metastasis is usually not seen until post-menopause, when collagen I and collagen III production slows.^18^

Our characterization of the ovary also revealed collagen III as a major structural component surrounding the CIC, where it was elevated in a pattern similar to the collagen I band. The incorporation of collagen III into a collagen I matrix has been shown to decrease the organization of collagen across several structural levels,^39^ which may result in a more permissive matrix for cell invasion. Indeed, when collagen III was added to the ECM in our model, FTE invasion was increased. These data suggest that imbalances in ECM turnover that increase the relative levels of collagen III could promote FTE cell invasion. While this is the first study to investigate cell invasion in collagen I/III gel mixtures *in vitro*, collagen III gels have previously been shown to increase endothelial cell angiogenesis compared to collagen I.^40^ Interestingly, decreasing the collagen I concentration increased both the number of invading cells and the average distance of invasion, while adding collagen III to the matrix surrounding lumens only increased the number of FTE cells that invaded. The collagen concentration and composition have varying effects on the collagen structure, which could explain the observation that less dense collagen creates larger pores in an organized matrix,^41^ while adding collagen III to the matrix disrupts collagen fiber organization.^39^ The flexibility of this biomimetic CIC model will permit additional perturbations of the ECM and soluble factors to be examined, in order to determine how elements of the CIC physical and chemical microenvironments influence the progression of metastatic FTE-derived cells to advanced HGSOC.

## METHODS

### Cell lines and reagents

Unless otherwise stated, all reagents were purchased from ThermoFisher (Waltham, MA). Mouse FTE cells with a *Tp53^R273H^* mutation were obtained from Dr. Joanna Burdette at the University of Illinois at Chicago.^42^ Mouse OSE cells were isolated from FVB mice under a protocol approved by the University of Wisconsin School of Medicine and Public Health Animal Use and Care Committee (Animal Committee No. M005940), following a previously established method.^43^ Cells were maintained at 37 °C in 5% CO_2_ in MEM (Minimal Essential Medium) Alpha Modification supplemented with 10% heat-inactivated fetal bovine serum (FBS), 2 mM l-glutamine (Sigma-Aldrich; St. Louis, MO), 2 ng/ml epidermal growth factor, 5 *μ*g/ml insulin (Roche; Basel, Switzerland), 5 *μ*g/ml transferrin (Roche), 5 ng/ml sodium selenite (Roche), 1.1 *μ*g/ml gentamicin (Corning; Corning, NY), and 0.055% penicillin/streptomycin.

### Collagen dot blot

Paraffin embedded samples of ovaries from women who underwent oophorectomy due to HGSOC or non-cancerous conditions were obtained from archived pathology samples through a protocol approved by the Institutional Review Board at the University of Wisconsin-Madison (IRB No. 2016–1152). Collagen hydrogels created from collagen I (Advanced Biomatrix; San Diego, CA) or collagen III (Advanced Biomatrix) were used as controls. From each sample, a 20 *μ*m section was cut and placed in a 1.5 ml tube and deparaffinization was performed by incubating with 1 ml of Safeclear II Xylene Substitute for 10 min at room temperature. The tissue and any undissolved paraffin were pelleted by centrifuging for 3 min at 16 000*g*. Safeclear washing and centrifuging were repeated two additional times until all the paraffin was dissolved and only tissue remained. The tissue pellet was rehydrated using serial dilutions of ethanol (100%, 70%, and 50%). The pellet was resuspended in 200 *μ*l of protein extraction buffer containing 20 mM Tris-hydrochloride (Promega; Madison, WI) and 2% sodium dodecyl sulphate (SDS, Boston Bioproducts; Ashland, MA), pH 8.0. The non-protein material was removed from the solution by centrifuging at 16 000*g* for 20 min at 4 °C. Collagen I and III levels were quantified by dot blot.^44^ Briefly, a polyvinylidene fluoride membrane (BioRad; Hercules, CA) was soaked in methanol for 1 min and then rinsed with water for 2 min. The membrane was then allowed to dry, and 1 *μ*l of standard and samples were pipetted onto the membrane. The membrane was incubated at 37 °C for 5 min to set the protein into the membrane, rinsed three times with tris-buffered saline (TBS, Boston Bioproducts), and blocked in TBS with 0.1% Tween-20 (TBST, Dot Scientific; Burton, MI) and 1% normal goat serum for 1 h at room temperature. Antibodies (anti-collagen I (ab34710, Abcam; Cambridge, United Kingdom) at 1:2000 and anti-collagen III (ab7778, Abcam) at 1:5000) were diluted in blocking solution and incubated with the blots overnight at 4 °C with agitation. The membrane was rinsed three times with TBST and incubated in goat anti-rabbit IgG H&L (HRP) secondary antibody (ab6721, Abcam) at 1:2000 in blocking buffer for 1 h. The membrane was then washed three times with TBST and three times with TBS. The blot was imaged using a Clarity Western ECL Substrate (BioRad) and an Odyssey Fc Imaging System (Licor; Lincoln, NE) and analyzed using FIJI software.^45^

### Immunofluorescence staining of ovary sections

From each paraffin-embedded ovary used for collagen dot blots, 10 *μ*m sections were cut and mounted on glass sides. The slides were deparaffinized with SafeClear II Xylene Substitute and rehydrated using serial dilutions of ethanol (100%, 95%, 70%) followed by two washes with TBST. Heat antigen retrieval was performed at 95 °C using Universal Antigen Retrieval Solution (R&D Systems; Minneapolis, MN) for 10 min, followed by a 10 min incubation at room temperature. Slides were washed twice for 5 min each in TBS supplemented with 1% bovine serum albumin (TBSB), and the tissue sections were encircled with a hydrophobic pen. The slides were then blocked in TBSB supplemented with 1% normal goat serum for 1 h at room temperature. Antibodies [anti-collagen I (ab34710, Abcam) at 1:100 and anti-collagen III (ab7778, Abcam) at 1:500] were diluted in the blocking solution and incubated on the sections at 4 °C overnight. Slides were washed four times in TBSB, and a goat anti-rabbit IgG H&L Alexa Fluor^®^ 488 secondary antibody (ab150077, Abcam, 1:300) was added for 1 h. Slides were rinsed three times with TBSB and sealed with ProLong^®^ Diamond Antifade Mountant with DAPI. Imaging was performed on a Zeiss Axio Observer.Z1 inverted microscope with an AxioCam 506 mono camera, a Plan-Neofluar 10× 0.4-NA air objective, a Plan-Apochromat 20× 0.8-NA air objective, and Zen2 software (Zeiss; Oberkochen, Germany).

### Quantification of the collagen concentration

FIJI was used to measure the total area of each ovary section, and this area was multiplied by the thickness of each section to obtain the tissue volume. The collagen I and collagen III concentrations from the dot blot assays were multiplied by the tissue resuspension volume (200 *μ*l) and divided by the tissue volume to obtain total collagen concentrations. To obtain the level of collagen in the cortex, the total collagen concentration was multiplied by the average fluorescence intensity of the cortex divided by the average fluorescence intensity of the whole ovary.

### Second harmonic generation imaging

The SHG imaging system consisted of a laser scanning head (Olympus FluoView 300, Olympus, Tokyo, Japan) mounted on an upright microscope (Olympus BX61), coupled to a mode-locked Titanium Sapphire laser. All measurements were carried out with a laser fundamental wavelength of 890 nm with an average power of ∼20 mW at the specimen. The microscope simultaneously collected both the forward and backward components of the SHG intensity. In the former, a long working distance 40 × 0.8 N.A. water-immersion objective and a 0.9 N.A. condenser provided excitation and signal collection, respectively. The backward component was collected through the excitation objective in a non-descanned configuration. In each channel, the SHG signal was isolated with a dichroic mirror and a 10 nm bandpass filter (445 nm). The signals were detected by two identical photon-counting photomultiplier modules (Hamamatsu 7421, Hamamatsu, Japan).

### Analysis of collagen alignment

CT-FIRE^46^ (Curvelet Transform followed by Fiber Extraction) was run on the z-stacks generated from second harmonic generation imaging to identify collagen fibers. CurveAlign^21^ was then used to identify each fiber's distance from and angle relative to a manually drawn CIC boundary. The fiber angle was binned into three groups with fibers with an angle between 0° and 30° classified as parallel, 30°–60° as random, and 60°–90° as perpendicular.

### Microfluidic devices

Microfluidic lumens were made using the LumeNEXT platform.^22^ Briefly, soft lithography was used to generate negative molds of the multilayer lumen device, and poly-dimethylsiloxane (PDMS, Dow Corning, Salzburg, MI) prepared at a 1 to 10 curing agent to elastomer base ratio was used to fabricate the top and bottom layers of the chamber. Linear PDMS rods with a 410 *μ*m diameter circular cross-section were created by filling 22G hypodermic needles with PDMS and removing the PDMS from the needles after curing. These circular lumen rods were inserted into the chamber, and the devices were bonded to a glass surface using oxygen-plasma treatment (Thierry Corp., Royal Oak, MI). The devices were sterilized by exposure to UV light for 20 min, pretreated with 2% poly(ethyleneimine) (Sigma) diluted in sterile deionized water for 10 min at room temperature followed by 0.1% glutaraldehyde (Sigma) diluted in sterile deionized water for 30 min at room temperature, and washed three times with sterile deionized water.

Collagen gel preparation was carried out on ice to halt polymerization of collagen. Bovine collagen type I (Advanced Biomatrix) was mixed with 1 part 10× serum free MEM Alpha Modification media, neutralized to a pH of 7.4 with 0.2 N sodium hydroxide (Sigma), and diluted to 10 parts with sterile deionized water. The mixture was incubated on ice for 20 min before use. By varying the initial concentrations, gels of 2.5, 4.8, and 7.7 mg/ml were formulated. The collagen gel mixture was loaded into each device, and a ring containing 200 *μ*l of phosphate buffered saline (PBS) was added around each device to prevent evaporation. The devices were incubated at room temperature for 10 min followed by overnight incubation at 37 °C. On the following day, 5 *μ*l of PBS was added to the small port of the lumen and the PDMS rod was pulled out of the polymerized collagen from the larger port, resulting in a lumen structure filled with PBS inside the collagen gel. The PBS was aspirated from each lumen, and 5 *μ*l of 1:10 Matrigel (Corning) diluted in serum-free media (SFM) was added to each lumen and incubated for 20 min at 37 °C. The lumens were washed once with SFM before seeding cells.

### Fluorescence labeling of collagen

To visualize collagen organization within microfluidic lumens, 5 *μ*l of collagen-binding adhesion protein 35 (CNA35-EGFP)^47^ at a concentration of 25 *μ*M was incubated in each lumen overnight at 37 °C. The lumen was then washed 5 times for 2 h each at 37 °C to remove unattached protein. The CNA35-EGFP plasmid was a gift from Maarten Merkx (Addgene plasmid # 61603).

### Cell seeding and culture in lumens

FTE and OSE cells were stained with 2 *μ*M CellTracker™ Green following manufacturer's directions, dissociated using trypsin-EDTA, and suspended in SFM at 100 000 cells/*μ*l. 4 *μ*l of cell suspension was added to each lumen through the small port, and the PBS ring surrounding the device was replenished. The lumens were rotated at 3 rpm for 2 h at 37 °C, washed once with 5 *μ*l of media to remove unattached cells, and incubated with 5 *μ*l of media per lumen. The devices were rotated at 3 rpm throughout the experiment, and the media in the lumens and the PBS ring around the device were changed every 12 h.

### Analysis of cell invasion

A Leica TCS SP8 confocal microscope (Leica; Wetzlar, Germany) was used to image cells in the lumens. Confocal slices at the vertical midpoint of the lumen were stitched together to visualize the entire length of the lumen and quantify invasion. The number of cells that invaded the collagen from the lumen and the distance invaded was measured in FIJI. Z-stacks were taken at a Nyquist sampling interval of 2.41 *μ*m to generate cross-sectional images of lumens. Rendering of z-stacks into three dimensional images was performed using Leica LAX software.

### Follicular fluid

Through a protocol approved by the Institutional Review Board at the University of Wisconsin-Madison (IRB No. 2016-1082, follicular fluid is classified as IRB exempt), human follicular fluid was collected at Generations Fertility Clinic (Middleton, WI) during *in vitro* fertilization procedures and frozen at −80 °C. Follicular fluid was collected from patients undergoing *in vitro* fertilization, which is currently the only procedure where this fluid can be obtained. It is unknown if the use of hormonal stimulation alters the composition of follicular fluid; however, follicular fluid is not retrieved for any other purpose besides egg collection for *in vitro* fertilization. Follicular fluid was thawed on ice and centrifuged at 1200*g* for 10 min to remove cell debris. Follicular fluid was then diluted to a final concentration of 6% in SFM,^48^ and 5 *μ*l of this solution was added to each lumen 2 h after seeding. SFM containing 6% PBS served as a vehicle control. As above, media in the lumens were changed every 12 h and lumens were rotated at 3 rpm for the duration of the experiment.

### Statistical analysis

Data are presented as mean ± standard deviation. Statistical calculations [two-sided t-test, paired t-test, one-way ANOVA (Analysis of Variance) followed by Tukey-HSD (Honest Significant Difference)] were performed using GraphPad Prism software (La Jolla, CA).

## SUPPLEMENTARY MATERIAL

See supplementary material for ovary pathology patient demographics, collagen I and collagen III dot blots, high magnification IMF images of blood vessels, chart of CIC diameters, and confocal image of OSE cells in our *in vitro* CIC model.
